# First Randomized Controlled Trial Comparing D-Mannose to Fosfomycin in Acute Uncomplicated Lower Urinary Tract Infections

**DOI:** 10.3390/antibiotics15090820

**Published:** 2026-08-24

**Authors:** Florian Wagenlehner, Heidrun Taeschner, Horst Lorenz, Anja Berwanger, Nacera Infed, Oda Ewald, Peter Gerke

**Affiliations:** 1 Clinic for Urology, Pediatric Urology and Andrology, Justus Liebig University Giessen, 35392 Giessen, Germany; florian.wagenlehner@chiru.med.uni-giessen.de; 2 Internistische Facharztpraxis, 04249 Leipzig, Germany; 3 BBS Neuberg, Büro für Biometrie und Statistik, Im Unterfeld 17, 63543 Neuberg, Germany; 4 MCM Klosterfrau Vertriebsgesellschaft mbH, Gereonsmuehlengasse 1-11, 50670 Cologne, Germany

**Keywords:** urinary tract infection (UTI), cystitis, D-mannose, antibiotics, acute cystitis symptom score, non-inferiority

## Abstract

**Background/Objectives**: Antibiotics are the recommended first-line therapy for acute uncomplicated lower urinary tract infections (cystitis), but antibiotic resistance and tolerability concerns require antibiotic-sparing strategies. This study evaluated D-mannose as a stand-alone alternative. **Methods**: This multicenter, randomized, controlled, double-blind study compared D-mannose to fosfomycin in women aged 18–70 with cystitis. Clinical cure (CC) was assessed using the Acute Cystitis Symptom Score (ACSS) up to day 8, with non-inferiority tested at the 15% margin. Post hoc analytical approaches accounted for potential bias due to low sample sizes and missing values. **Results**: The study randomized 118 patients. At baseline, severe symptoms (ACSS ≥ 12) were more frequent in the D-mannose group compared to fosfomycin (32.8% vs. 22.8%). Efficacy analyses were performed on the per-protocol set (D-mannose: N = 57; fosfomycin: N = 54). Median time to CC was 5.0 days (D-mannose) and 3.0 days (fosfomycin) overall, and 4.0 days vs. 3.0 days in the subgroup of moderate disease severity. The point estimate for the CC rate difference on day 8 (−10.1%) favored fosfomycin but was within the non-inferiority margin. Due to wide confidence intervals, non-inferiority was not statistically confirmed. The post hoc analysis yielded smaller confidence intervals with estimated CC rates of 84.2% (D-mannose) vs. 83.5% (fosfomycin) on day 8 and statistically demonstrated the comparability of both treatments. Recurrence rates were similar (13.2% vs. 13.3%), and there was no statistically significant difference for additional antibiotic use (19.3% vs. 11.1%). Investigators and patients favored D-mannose over fosfomycin for tolerability, and gastrointestinal adverse events were less frequent (9.8% vs. 24.6%). **Conclusions**: Post hoc analysis indicated that D-mannose treatment is comparable to fosfomycin in uncomplicated cystitis in line with the prespecified non-inferiority criteria. These findings warrant confirmation in an adequately powered trial but suggest that D-mannose is a promising alternative to antibiotics given its favorable risk–benefit ratio.

## 1. Introduction

Acute uncomplicated infections of the lower urinary tract are common in the outpatient setting. Risk factors include female sex, sexual intercourse, and vaginal infection [1]. The lifetime prevalence of urinary tract infections (UTIs) among women is approximately 40%, and recurrent episodes of UTI occur in one in five women [2].

Most cases of acute uncomplicated UTIs (hereafter referred to as cystitis unless otherwise noted) are caused by bacterial pathogens, primarily uropathogenic *Escherichia coli* (*E. coli*), which accounts for 75% of all cases [3]. Diagnosis of acute cystitis does not require microbiological confirmation and, in the absence of vaginal discharge, can be made on the basis of clinical symptoms alone, i.e., dysuria, frequency, and urgency [4]. Dipstick urinalysis for blood, leukocytes, and nitrite can increase diagnostic accuracy, especially in equivocal cases [4]. The Acute Cystitis Symptom Score (ACSS), a validated questionnaire with high sensitivity and specificity, enables the symptom-based diagnosis and subsequent monitoring of the infection [5].

The European Association of Urology (EAU) guidelines on urological infections recommend oral antibiotics, namely fosfomycin trometamol, pivmecillinam, or nitrofurantoin, as first-line treatment for cystitis [4]. The German guideline in addition recommends nitroxoline and trimethoprim [5]. Fosfomycin, a broad-spectrum antibiotic that disrupts cell wall synthesis [6], achieves a clinical cure (CC) rate of 78.7% in cystitis with a single dose according to a post hoc calculation [7]. Although generally well tolerated, gastrointestinal side effects such as diarrhea and nausea are common [8], likely due to imbalances in the gut microbiome [9]. The global burden of antibiotic resistance also affects the treatment of cystitis [10]. According to the current guidelines, non-antibiotic options such as ibuprofen and certain phytotherapies can be considered in non-geriatric patients or patients with mild to moderate symptoms as primary therapy as part of a symptomatic approach following participatory decision-making [4,5].

An example of an effective and causal non-antibiotic strategy is the disruption of bacterial adhesion to the urinary tract epithelium, a key step in infection [11]. Glycoproteins serve as adhesive structures for uropathogenic bacteria with so-called type 1 fimbriae [12]. D-mannose, a natural glucose epimer, saturates these bacterial adhesive structures, namely FimH, and prevents bacterial attachment to bladder cells [13,14]. 

Several studies have demonstrated the efficacy of D-mannose, alone or in combination, for the prevention of recurrent UTI and provided preliminary support for its use in acute treatment [15,16,17,18,19,20]. A non-interventional study (NIS) in acute cystitis was performed with D-mannose as monotherapy [21], and a post hoc analysis of this NIS compared D-mannose with antibiotics, showing promising potential as an alternative treatment [22]. However, direct, high-level, comparative evidence against antibiotics was still limited. Therefore, the present randomized controlled clinical trial was designed to confirm the safety and efficacy of D-mannose as a stand-alone treatment for acute cystitis as compared to antibiotic first-line therapy.

## 2. Results

### 2.1. Study Population

The clinical study (EudraCT 2021-003466-12) was conducted from May 2022 to August 2023 in 22 medical practices and clinical trial units in Germany. The trial ended as planned. By July 2023, a total of 134 adult female outpatients with symptoms of acute cystitis had been screened and 118 patients randomized. The full analysis set (FAS) and the safety evaluation population (SEP) consisted of 61 patients randomized to the D-mannose group and 57 patients randomized to the fosfomycin group. Of these, 25 patients discontinued the study prematurely, mainly due to the need for additional antibiotic therapy for cystitis (Supplementary Figure S1). The per-protocol set (PPS) included 57 patients in the D-mannose group and 54 patients in the fosfomycin group. Reasons for exclusion from the FAS were missing visits (five patients), violation of a key exclusion criterion (one patient), and prohibited concomitant medication (one patient).

### 2.2. Patient Characteristics

Patient demographic and clinical characteristics were well balanced, with no significant differences between the two treatment groups (Table 1). Similar values were obtained for the PPS (Supplementary Table S1).

### 2.3. Clinical Cure (CC)

**Figure 1:**
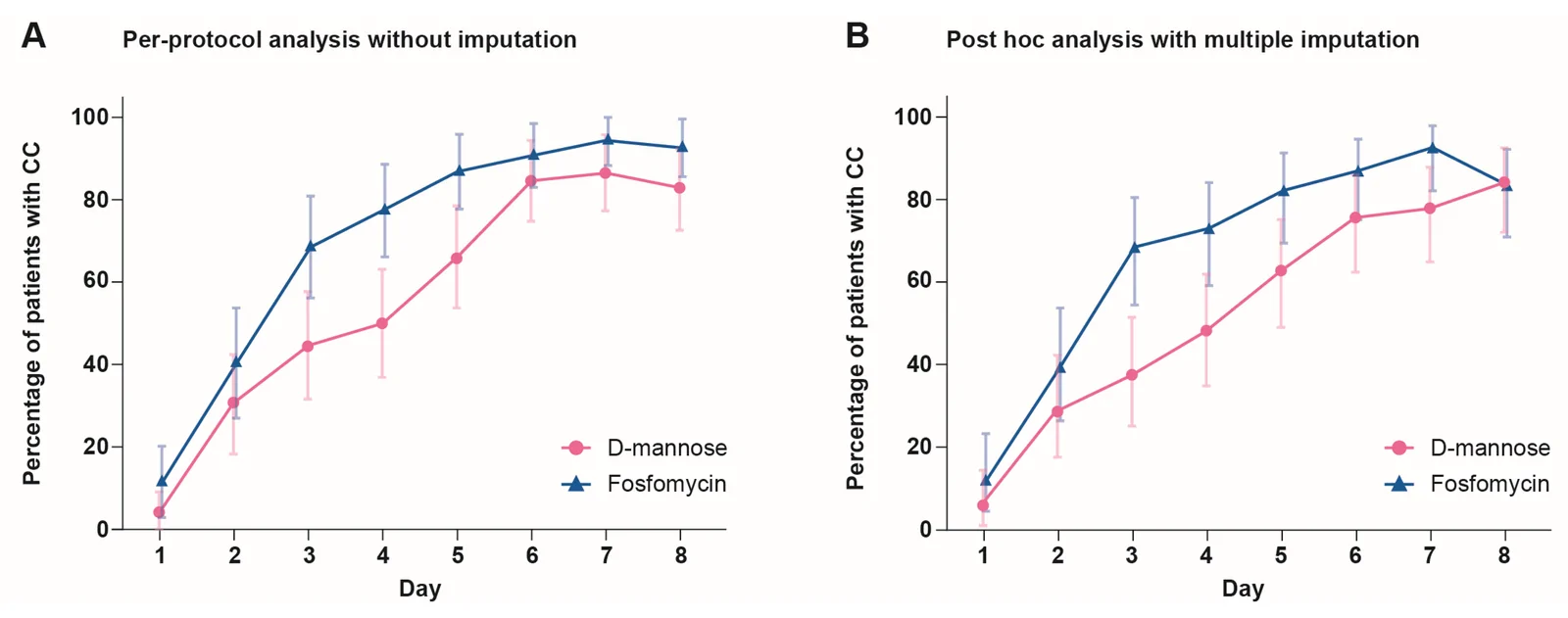
Time course of clinical cure (CC) rates (main definition, i.e., ACSS Typical Domain Score ≤4 including all items) up to day 8 according to the per-protocol analysis (**A**) and the post hoc analysis after applying multiple imputation procedures (**B**). Markers indicate CC rate estimates, error bars indicate 95% CIs. Analyses were performed on the per-protocol set (PPS).

According to the per-protocol analysis, on day 8, the CC rate according to the main definition (ACSS Typical Domain Score ≤4 including all items) was lower in the D-mannose group (82.5%; 47/57 patients) than in the fosfomycin group (92.6%; 50/54 patients) (Figure 1A). Results were similar for the CC definition variation 1 (ACSS Typical Domain Score ≤4 excluding the item ‘incomplete voiding’) and the CC definition variation 2 (ACSS Typical Domain Score ≤3 excluding the items ‘incomplete voiding’ and ‘suprapubic pain’). Namely, the proportion of patients with CC was lower in the D-mannose group compared to the fosfomycin group in variation 1 (82.5% vs. 94.4%) and variation 2 (84.2% vs. 94.4%). Sensitivity analysis of the endpoint clinical cure at Day 8 using the FAS are in line with the PPS results (Figure S2). The difference in CC rates (point estimate −10.1%, main definition) on day 8 did not exceed the non-inferiority threshold of −15.0%. However, non-inferiority could not be demonstrated at this point due to wide confidence intervals. The proportion of patients with CC (main definition) increased in both treatment groups until day 29. The difference in CC rates (−4.0%) on day 29 between the D-mannose group and the fosfomycin group (89.8%; 44/49 patients vs. 93.8%; 45/48 patients), as well as the lower limit of the 95% CI (−14.9, 6.9), did not exceed the −15% margin, and therefore the criterion for non-inferiority was met.

Per-protocol time-to-event analyses showed that patients in the D-mannose group achieved CC two days later than patients in the fosfomycin group (median time to CC 5.0 days vs. 3.0 days). In patients with moderate disease severity at baseline (ACSS Typical Domain Score 6–12), the difference between the groups was reduced to one day (median time to CC 4.0 days vs. 3.0 days). Patients with severe disease at baseline (ACSS Typical Domain Score ≥ 12) reached CC after a median of 6.0 days (D-mannose) and 3.0 days (fosfomycin). Of note, rapid improvement in symptoms indicating considerable healing progress was observed days before the definition of CC was reached (see Section 2.4). This early onset of symptom improvement possibly weighs more in patients’ perception than achieving formal CC (see Section 2.7). The proportion of patients who achieved CC within the first eight days according to the time-to-event analyses in the total population (86.0% vs. 94.4%) was consistent with the analysis of CC rates as described above. The minor differences between the approaches can be attributed to the respective evaluation schedules. The rate-based approach considered only the CC rates on day 8 specifically, whereas the time-to-event approach analyzed the first occurrence of CC at any time up to day 8.

Post hoc analysis corrected for potential bias due to missing values and the persistence rule (i.e., per-protocol CC persisted until recurrence of UTI or the last day of the study). To reduce this bias, a multiple imputation procedure was applied to the data, and the persistence rule was disregarded. This approach resulted in estimated CC rates of 84.2% for D-mannose and 83.5% for fosfomycin on day 8. Estimates over time showed strong improvement from day 1 to day 8 in both groups, with an approximately linear course from day 1 to day 8 in the D-mannose group. In the fosfomycin group, the increase in CC rates was initially greater up to day 3 and less thereafter. From day 6 on, the curves converged considerably, indicating a reduced difference between the groups (Figure 1).

**Figure 2:**
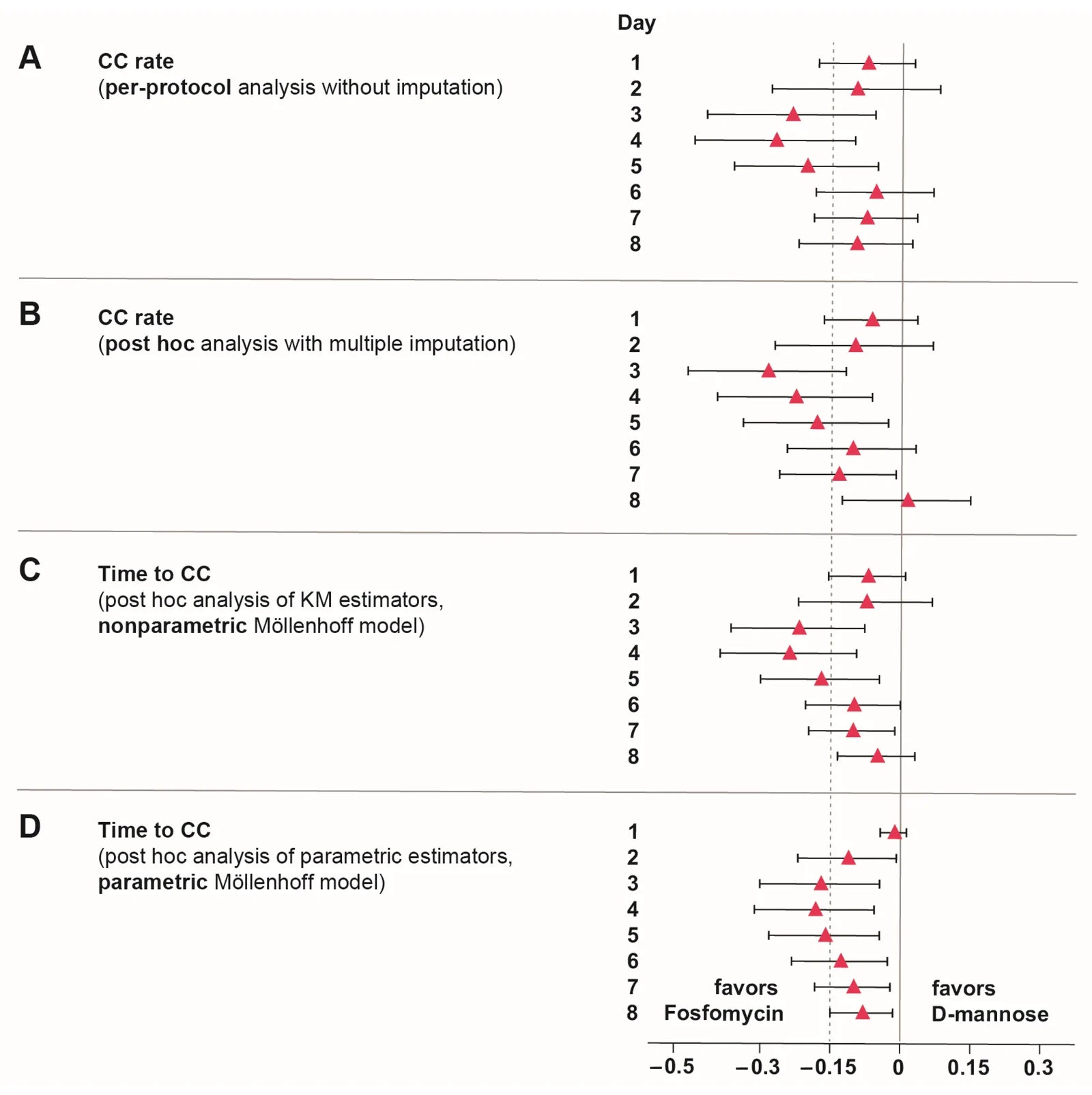
Non-inferiority assessments for treatment differences in clinical cure (CC) from day 1 to day 8 in the per-protocol set (PPS) based on the per-protocol analysis of CC rates (**A**), based on the post hoc analysis of CC rates after multiple imputation (**B**), or based on the post hoc analysis of time to CC applying either Kaplan–Meier (KM) estimates (nonparametric Möllenhoff model) (**C**) or parametric estimators (parametric Möllenhoff model) (**D**). Red markers indicate point estimates of treatment differences, bars indicate 95% CI. The dashed line indicates the non-inferiority margin.

Post hoc analysis of time-to-event evaluations were based on the CC data of the per-protocol analysis and the time of first occurrence of CC without (Figure 2A) and after (Figure 2B) multiple imputation of the ACSS Typical Domain Scores. Furthermore, the time-to-event estimators for single time points were based on post hoc models as proposed by Möllenhoff and Tresch [23], applying the standard Kaplan–Meier (KM) technique (Figure S3A) and a parametric structure (Figure S3B). This statistical technique enabled use of a difference scale even for time-to-event models. Consequently, the same non-inferiority margin (15%) could be used for both CC endpoints (CC rate and time to CC).

The results were consistent with the per-protocol analyses with regard to median time-to-event (D-mannose: 5 days; fosfomycin: 3 days). Multiple imputation in the rate-based approach (basic model) slightly altered the non-inferiority assessments of the treatment differences in CC from day 1 to day 8 (Figure 2A,B). The precision of the conclusion increased, as indicated by the steady decrease in confidence interval lengths from the basic model (Figure 2B) to the nonparametric Möllenhoff model (Figure 2C) and parametric Möllenhoff model (Figure 2D). Therefore, the parametric model was considered to be a more adequate description of the time trends reported.

All four approaches indicated a treatment difference in favor of fosfomycin from day 3 to day 5 only. But thereafter this difference clearly narrowed, and the point estimators were above the defined non-inferiority margin of −15.0% (Figure 2A–D). On day 8, comparability of D-mannose to fosfomycin was statistically demonstrated given that the lower boundary of the confidence interval was within the limits of the predefined non-inferiority margin in all the post hoc analytical approaches (Figure 2B–D).

**Figure 3:**
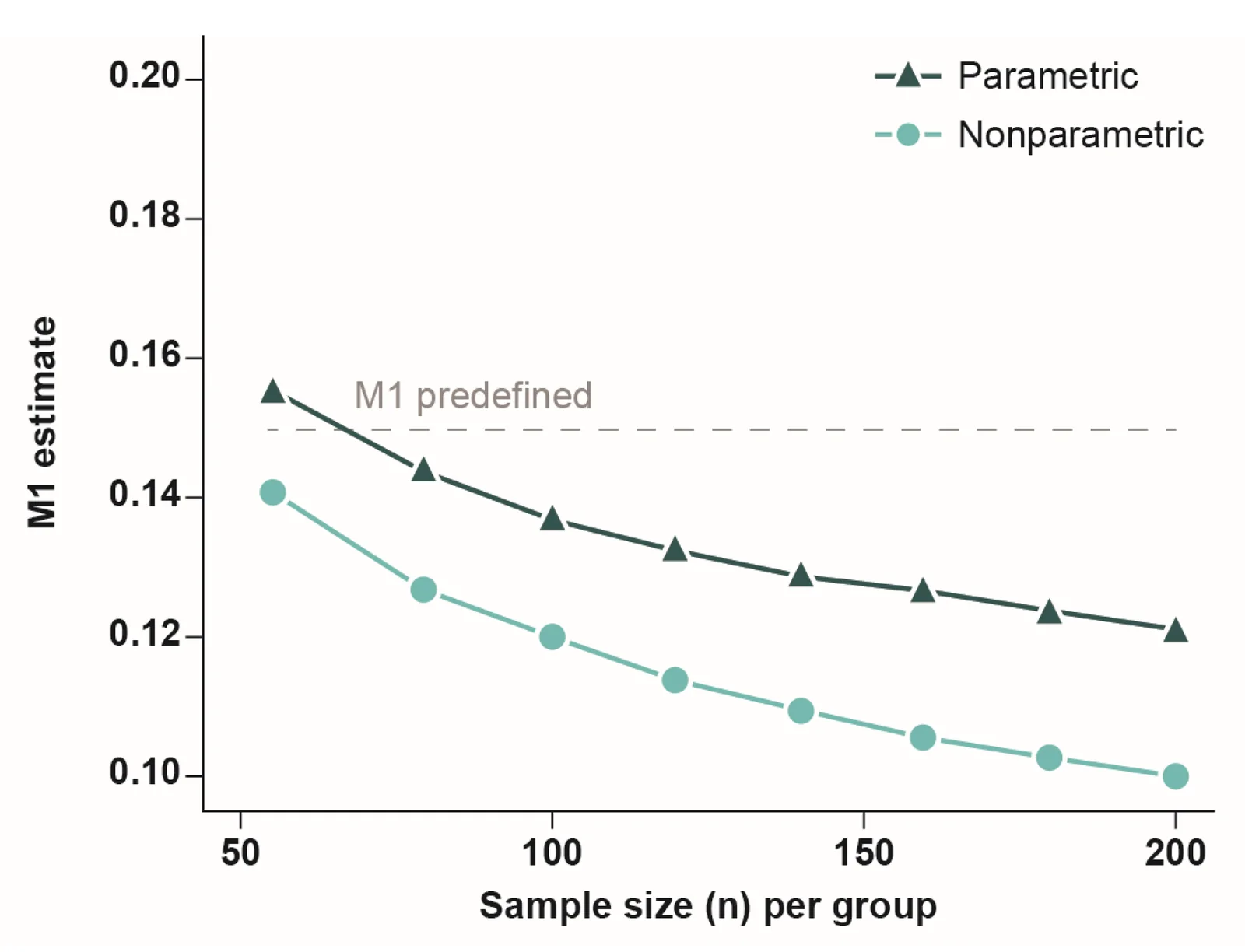
Simulation of the sample sizes required to demonstrate non-inferiority for different treatments with regard to CC (main definition, i.e., ACSS Typical Domain Score ≤4 including all items). Non-inferiority estimates (M1) are presented by group sample sizes for day 8 based on the nonparametric Möllenhoff model and the parametric Möllenhoff model using study data from the per-protocol set (PPS). Markers indicate the M1 estimates. The dashed line (M1 predefined) indicates the pre-specified non-inferiority margin of 0.15.

Based on the meaningful results of the post hoc imputation and modelling approaches, post hoc simulations were performed to estimate the sample size required to demonstrate non-inferiority in future trials (Figure 3). The simulations applied both Möllenhoff approaches with CC on day 8 as an efficacy parameter due to its clinical relevance (main definition as defined per protocol without the persistence rule). Accordingly, non-inferiority estimates (M1) < 0.15, i.e., a situation in which the difference between test and comparator treatment including 95% CI does not exceed 15% in favor of the comparator, might be achieved with high probability even with sample sizes of n = 50 per group for the nonparametric Möllenhoff KM model and with sample sizes of at least n = 80 per group for the parametric Möllenhoff model. To sum up, the parametric Möllenhoff model is anticipated to generate more precise estimates due to its relative complexity, especially with small sample sizes.

### 2.4. Time Course of ACSS Typical Domain Score

**Figure 4:**
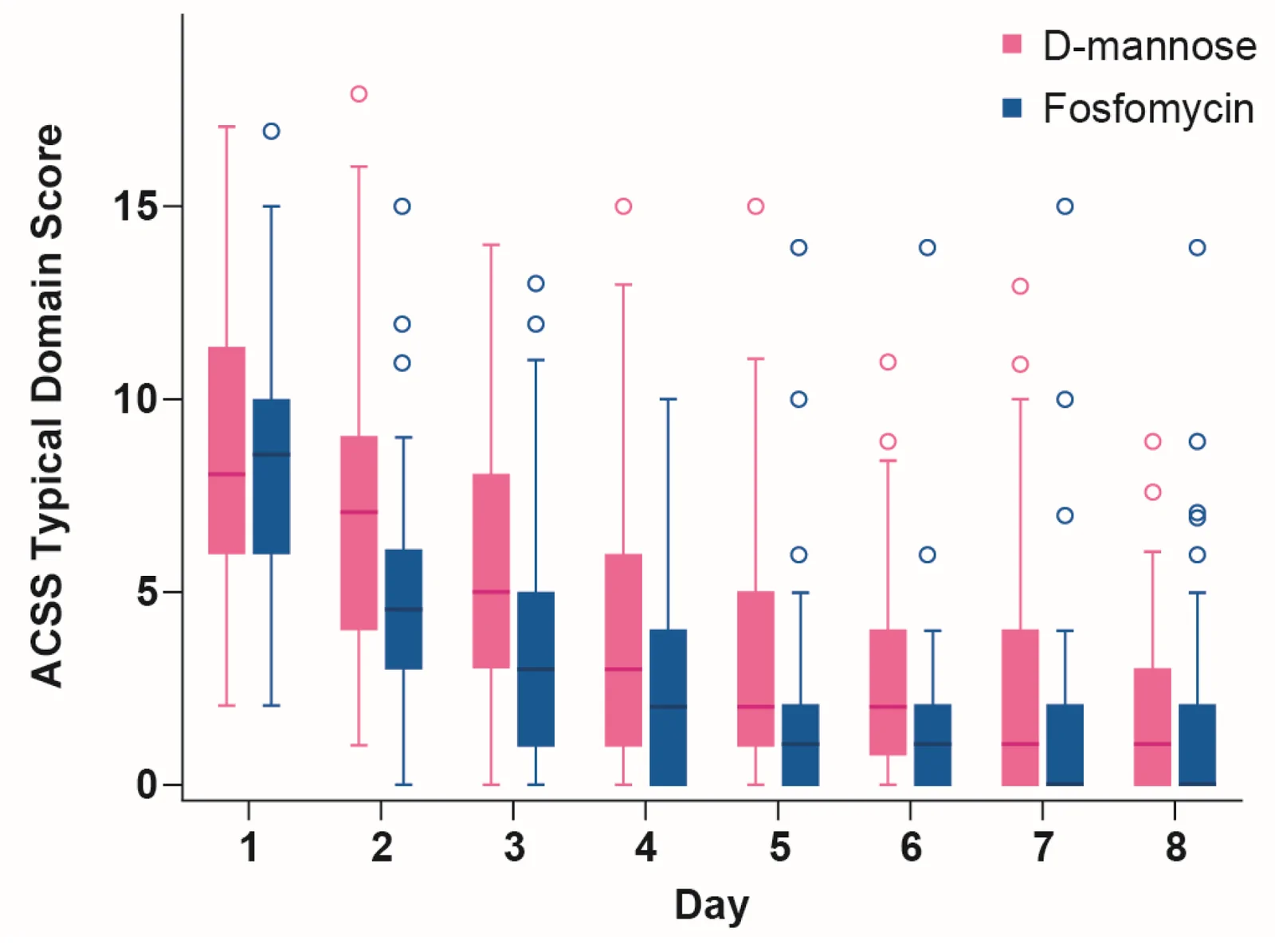
Post hoc analysis of ACSS Typical Domain Score over time after multiple imputation in the per-protocol set (PPS). Boxes indicate 25% to 75% quartiles; lines indicate the medians. Whiskers are maximum 1.5 times the interquartile range (IQR) in length. Outliers (>1.5 × IQR) are indicated by colored circles.

The proportion of patients with more severe symptoms at baseline was greater in the D-mannose group compared to the fosfomycin group in the PPS (31.6% vs. 24.1%, Supplementary Table S1). Despite this disbalance, both treatments showed a strong and rapid improvement of symptoms according to the per-protocol analysis of the ACSS Typical Domain Score (Supplementary Figure S4). The post hoc analysis with multiple imputation of missing values is consistent with the per-protocol analysis. Accordingly, in both groups, symptom severity was reduced by at least 80% from day 1 to day 8. The reduction in the fosfomycin group was stronger over the first few days, but the courses converged towards day 8, indicating comparable symptom relief in both groups overall (Figure 4). No further differences between the groups were observed in the follow-up phase from day 9.

### 2.5. UTI Recurrence and Antibiotic Treatment

During the follow-up phase, the incidence of UTI recurrence (defined as an ACSS Typical Domain Score ≥6 in patients who were previously in CC) was 12.3% (7/57 patients) in the D-mannose group and 13.0% (7/54 patients) in the fosfomycin group. However, unequal overall dropout rates (14.0%, 8/57 patients vs. 5.6%, 3/54 patients) necessitated a post hoc analysis to adjust for the number of dropouts. This analysis estimated a mean recurrence rate of 13.2% in the D-mannose group and 13.3% in the fosfomycin group, supporting similar recurrence rates in both groups. Additional antibiotic treatment was required at various times up to day 29 in 11 patients (19.3%) in the D-mannose group compared to 6 patients (11.1%) in the fosfomycin group. The rate was not significantly different for the two groups (rate difference: 8%; 95% CI: −6, 21).

### 2.6. Microbiological Status

The proportion of patients with a negative urine culture on day 8 was lower in the D-mannose group than in the fosfomycin group (57.4%, 31/54 evaluable patients vs. 66.7%, 34/51 evaluable patients), but the difference did not reach statistical significance (*p* = 0.311; normal approximation of the difference applying an arcsine transformation). Among patients with a positive urine culture at baseline (D-mannose: N = 34, fosfomycin: N = 33; PPS), 39.4% (13/33 evaluable patients) in the D-mannose group vs. 59.4% (19/32 evaluable patients) in the fosfomycin group had a negative urine culture on day 8. The difference in microbiological success rate was −20.0% (95% CI: −43.8, 3.8).

### 2.7. Global Assessment of Efficacy

The efficacy of treatment was rated either ‘very good’ or ‘good’ by the majority of investigators (66.7%, 38/57 patients vs. 75.9%, 41/54 patients) and patients (70.2%, 40/57 patients vs. 79.6%, 43/54 patients) in the D-mannose and the fosfomycin group, respectively. Overall, this suggests high treatment satisfaction with only minor differences between the groups despite the delay in achieving CC (Supplementary Figure S5).

### 2.8. Safety Results

Treatment-emergent adverse events (AEs) occurred in 32.8% of patients in the D-mannose group and 45.6% of patients in the fosfomycin group. All AEs were mild to moderate in intensity, with one exception. A total of 2.5% of patients discontinued treatment and withdrew from the study due to AEs. The incidence of drug-related AEs was distinctly higher in the fosfomycin group (22.8%) compared to the D-mannose group (9.8%). The most affected System Organ Class was gastrointestinal disorders with an AE incidence of 9.8% in the D-mannose group compared to 24.6% in the fosfomycin group, with diarrhea occurring in 1.6% and 21.1% of the SEP, respectively. Group comparisons revealed a statistically significant difference in the occurrence of gastrointestinal disorders in favor of D-mannose. A small *p*-value (*p* < 0.08) in the group comparison of AEs related to the study drug indicated a trend towards better tolerability of D-mannose compared to fosfomycin overall (Table 2).

**Figure 5:**
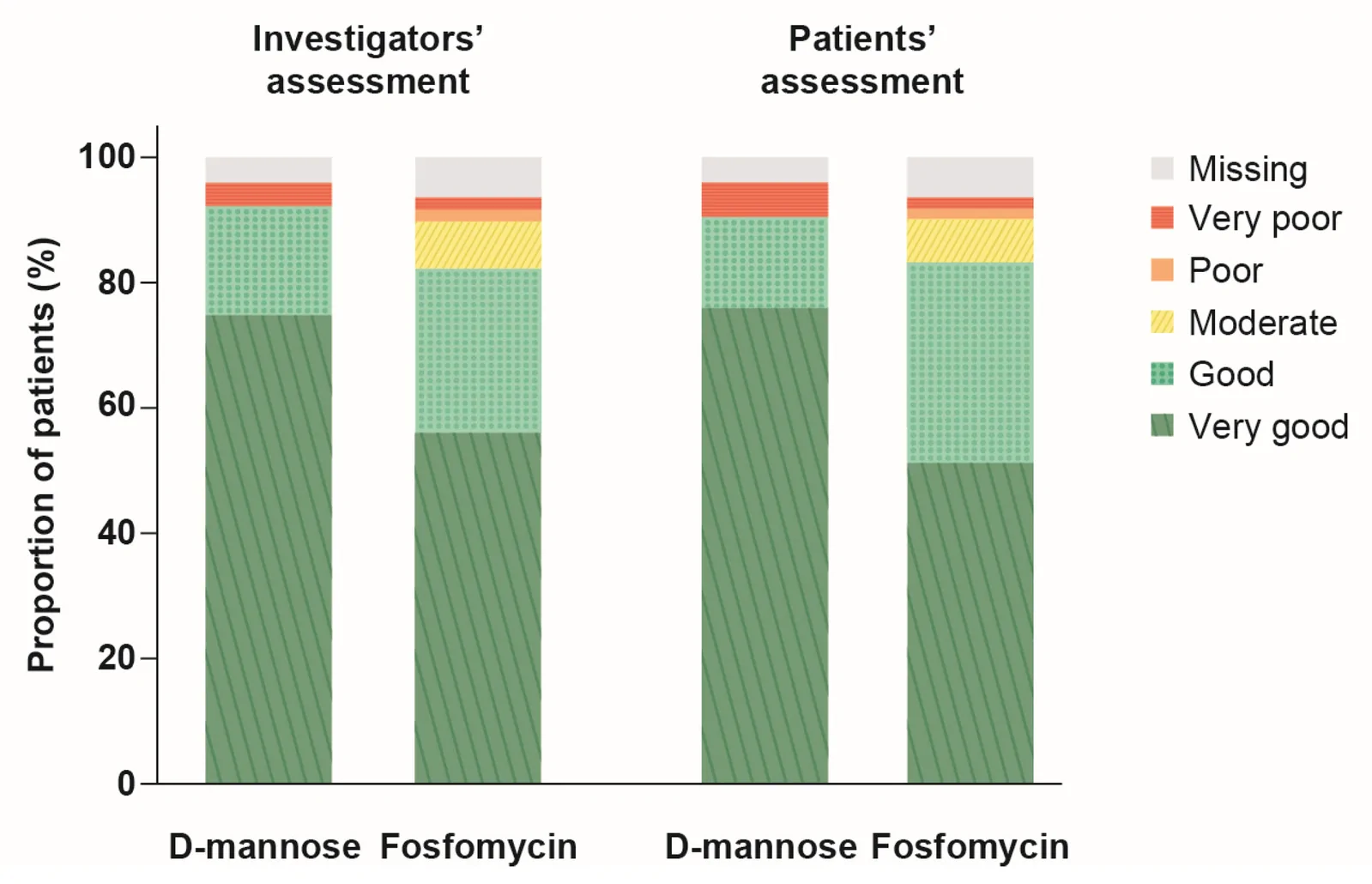
Global assessment of tolerability on day 8 in the safety evaluation population (SEP). Missing data at visit 2 was imputed with the worst category ‘very poor’ in case of discontinuation due to intolerable AEs. All other missing data are summarized under the category ‘missing’.

On day 8, tolerability was rated ‘very good’ or ‘good’ by 91.8% of investigators (56/61 patients) for D-mannose and 82.5% (47/57 patients) for fosfomycin, with a statistically significant advantage for D-mannose (*p* = 0.0456) (Figure 5). Patients’ tolerability ratings were similar (90.2%, 55/61 patients vs. 82.5%, 47/57 patients; *p* = 0.0075). ‘Very good’ tolerability was reported more frequently for D-mannose than for fosfomycin (investigator ratings 73.8%, 45/61 patients vs. 56.1%, 32/57 patients; patient ratings 75.4%, 46/61 patients vs. 50.9%, 29/57 patients). The proportion of patients with ‘poor’ or ‘very poor’ tolerability was similar in both treatment groups on day 8. Day 29 assessments showed similar results.

## 3. Discussion

The present proof-of-concept study indicates comparable efficacy of D-mannose monotherapy in females with cystitis compared to antibiotic first-line therapy with fosfomycin, using a non-inferiority design and post hoc analysis. Both treatments achieved high CC rates on day 8 with a difference in median time to CC of two days (5.0 vs. 3.0 days). In patients with typical symptom severity at baseline, the difference in time until CC was reduced to one day (4.0 vs. 3.0 days). Due to the small sample size, which led to wide confidence intervals, non-inferiority with a margin of 15% could not be confirmed in the per-protocol analysis, but a trend was apparent. A post hoc analysis that accounted for the small sample size showed CC rates of 84% on day 8 for both groups and statistically demonstrated comparability. Furthermore, although the proportion of patients with severe symptoms at baseline was higher in the D-mannose group (32.8% vs. 22.8%), the time course of symptoms demonstrated strong and rapid improvement under both treatments, emphasizing the comparable efficacy of both. In patients with positive culture at baseline, a lower microbiological success rate (13/33 vs. 19/32) was observed. However, the estimated recurrence rates during the follow-up phase were low and almost identical in both arms (13.2% vs. 13.3%). A higher need for additional antibiotic treatment throughout the study (19.3% vs. 11.1%) was observed in the D-mannose group, but this difference did not reach statistical significance. Furthermore, these results indicate that in 80% of patients in the D-mannose group, antibiotic treatment could completely be spared. Despite the observed difference in time to cure of two days or one day, respectively, only 10% of patients were less satisfied with the efficacy of D-mannose compared to fosfomycin, according to the global assessment. The global assessment of tolerability by investigators and patients favored D-mannose over fosfomycin and the incidence of gastrointestinal adverse events was substantially reduced with D-mannose treatment (9.8% vs. 24.6%), in particular the frequency of diarrhea (1.6% vs. 21.1%).

This non-inferiority study was designed with a significantly higher degree of standardization than previously published studies on D-mannose in this indication. The present study is therefore well suited to provide valid evidence for the efficacy of D-mannose and fosfomycin in the treatment of cystitis. The high level of standardization is reflected in the treatment allocation per randomization, the choice of comparator, the statistical approach, the choice of endpoints, and the precise definition of the study population. The key selection criteria focused on the severity and limited duration of clinical signs before study entry, without restriction to patients with a positive urine culture. Therefore, the study population represents standard clinical cystitis cases very well.

The concept of D-mannose as an appropriate treatment for cystitis was based on preliminary evidence from several trials, which not only showed that D-mannose monotherapy could effectively protect against UTI recurrence [15,16,17,19], but also suggested symptom improvement in acute therapy [15]. A non-interventional study then provided evidence for the effectiveness of D-mannose monotherapy in cystitis with 78.6% patients free of cystitis symptoms up to day 7 [21]. In comparison, the spontaneous healing rate has been reported to be up to 50% after one week [5]. Of note, the time and definition of CC assessment varies between clinical trials in this indication but mostly lies within a range of ±1 day in comparison to the present study, which used day 8 for assessment of CC.

Fosfomycin was selected precisely because it is highly efficacious, is recommended for first-line treatment, and has the highest possible compliance rate due to its single dose use [4], all of which makes it the most appropriate antibiotic comparator. Cure rates of 78% for CC and 79% to 84% for microbiological cure have been estimated for fosfomycin from two meta-analyses [22], and while the estimates showed no significant differences from other antibiotics, another recent meta-analysis concluded that fosfomycin might be more efficacious than other first-line antibiotics [24]. The fosfomycin results from the present study are consistent with previously reported estimates. The CC rates achieved with D-mannose are also in line with previously reported data [21,22], and the ACSS Typical Domain Score showed a similar time course, with a decline starting from the first day of treatment and a reduction of approximately 80% in median symptom scores by the end of the observation period [22].

Another non-antibiotic treatment investigated in a similar randomized clinical non-inferiority trial is the phytotherapeutic Canephron^®^ (BNO 1045), which was likewise shown to have antibiotic-sparing potential. It was found that 83.5% of patients treated with Canephron^®^ and 89.8% of patients treated with fosfomycin did not require additional antibiotic therapy. Although the treatment effect estimate met the non-inferiority criterion [25], the claim was based on antibiotic sparing as a surrogate endpoint for clinical efficacy instead of a hard CC endpoint. It is worth noting that the Canephron^®^ study included approximately 300 patients per group, which exceeds the size of the present study. It also exceeds the current simulation results for D-mannose, which indicate a sample size of at least 80 per group to demonstrate non-inferiority of D-mannose to fosfomycin in future studies applying a direct efficacy endpoint. The microbiological success rate of fosfomycin treatment was lower in the Canephron^®^ study (36.7%) than in the present study (59.4%). Given the similar CC rates in both studies, the inconsistent microbiological results call into question the accuracy and clinical relevance of the microbiological endpoints and therefore their suitability for non-inferiority assessment [7]. This also applies to the combined endpoint of clinical and microbiological cure as recommended by the European Medicines Agency (EMA) guidelines on clinical trials in UTIs [26]. Clinical trials routinely apply cut-off levels at baseline of 10^3^ CFU/mL or higher for patients with a positive urine culture despite the presence of clinical symptoms [25,27], leaving up to 40% of patients unevaluable for (combined) microbiological endpoints. Finally, in clinical routine, microbiological assessments are interpreted in the context of clinical symptoms. They are impacted by—and therefore dependent on—clinical symptoms, which calls into question endpoints that combine clinical and microbiological cure. These aspects further underline the lack of suitability of both the microbiological endpoint and combined clinical and microbiological endpoint for assessments of efficacy. Consequently, a purely clinical assessment of cure based on a validated symptom score, i.e., the ACSS Typical Domain Score, is unambiguous and no less suitable than the combination of symptom burden and microbiological examination [28].

The present study uses the ACSS Typical Domain Score as a patient-reported outcome measure (PROM) for diagnosis at enrolment, assessment of clinical course, and definition of CC. This is appropriate because the ACSS assesses the typical symptoms of acute cystitis and has been shown to be an effective diagnostic and monitoring tool [5,7,28,29]. It is compatible with the diagnostic criteria set out in the current guidelines of the US Food and Drug Administration (FDA) and the EMA guidelines [22,28]. The suitability of the ACSS for clinical trials has been previously evaluated [7,28,29,30,31] and symptom-based diagnosis is recommended in the current disease management guidelines [4,5]. Furthermore, the consistency of CC rates and recurrence rates from the present study in comparison to other studies suggest that a purely symptom-based approach is accurate. The efficacy endpoints based on the ACSS Typical Domain Score therefore enable valid conclusions on the suitability of D-mannose monotherapy for uncomplicated cystitis.

The benefits of antibiotic use are indisputable, but the increasing prevalence of antibiotic resistance calls for restrictions on their use. Although the resistance rates of *E. coli* to fosfomycin and nitrofurantoin are reported to be low, resistance of uropathogens to other typical antibiotics is observed in more than 20% of cases. For trimethoprim the rate is even higher [32]. It follows that antibiotic resistance is also an issue in the treatment of UTI and the use of antibiotics should wherever possible be avoided. Furthermore, fosfomycin has attracted particular attention as an effective treatment against multidrug-resistant pathogens. Resistance to fosfomycin could threaten its role in this context should resistance spread [6]. This is why the EMA has issued recommendations to restrict its use [33]. Although these restrictions do not directly affect the oral use of fosfomycin for cystitis in women, they do highlight the need for a cautious approach to antibiotic therapy and for alternative treatment options. In addition, tolerability issues affect the use of antibiotics in the treatment of cystitis, with gastrointestinal side effects such as diarrhea or nausea frequently reported [8] and attributable to antibiotic-induced imbalances in the gut microbiome [9]. These disruptions may contribute to increased rates of infection, immune dysregulation, and metabolic disorders. Furthermore, the gut microbiome has been discussed as a possible factor in the development of antibiotic resistance [9].

According to the German guideline, non-antibiotic therapies should be considered as an alternative to antibiotic therapy in non-geriatric patients following participatory decision-making [5]. However, the guideline emphasizes that the primary non-antibiotic treatments mentioned may be associated with a higher symptom burden and higher complication rates in pyelonephritis than observed with antibiotics. For these non-steroidal, anti-inflammatory substances (ibuprofen, diclofenac) and phytotherapeutics (uva-ursi, BNO 1045), the guideline shows an average saving of 63% on antibiotics in acute therapy. According to the present study, D-mannose has the potential for avoiding antibiotics in acute therapy in at least 80% of patients. The study also showed that although this substantial antibiotic-sparing effect of D-mannose was associated with a longer time to CC, no complications and fewer AE were observed with D-mannose, and the overall therapeutic outcome including symptom relief is presumably comparable to that of fosfomycin.

Despite meeting stringent study design criteria as outlined above, the results of the present study are subject to certain limitations. The relatively small sample size, due to slow recruitment, reduced the statistical power of the study. As a sample size of 50 per group was estimated to be sufficient for the proof-of-concept status of the study, the confidence intervals were rather wide and testing in the per-protocol analysis could, therefore, not confirm non-inferiority statistically, despite clear trends. A post hoc analysis reduced the width of the confidence intervals and generated a valid estimate of the efficacy of D-mannose. The state-of-the-art statistical methods used in this analysis to account for the small sample size are robust and appropriate. Accordingly, the post hoc analysis demonstrated comparability applying the prespecified non-inferiority criteria in the key endpoint of CC rate and congruent progress in symptom relief based on the ACSS Typical Domain Score. Taken together, the overall trend observed and the post hoc demonstration of comparability are sufficient to consider D-mannose therapy as a medically relevant alternative to antibiotic treatment. Furthermore, the simulations showed that future pivotal studies would be successful with a sample size of at least 80 patients per group.

In conclusion, post hoc analysis of the present study indicates that D-mannose is an effective and well tolerated treatment for cystitis. It is reasonable to consider D-mannose to be comparable to antibiotics in terms of CC rates and symptom relief, while maintaining a superior risk profile, particularly in terms of adverse effects such as diarrhea. As a result, it presumably offers a favorable risk–benefit ratio, making it a promising therapeutic option, particularly in self-medication and in patients who are reluctant to undergo antibiotic therapy. Larger confirmatory trials will be needed to establish D-mannose as an equivalent alternative to antibiotics.

## 4. Materials and Methods

### 4.1. Study Design and Conduct

MCMK0220 was a phase IV, multicenter, randomized, double-blind, double-dummy, active-controlled, two-arm, parallel-group study. It was designed as a proof-of-concept study to compare the efficacy, safety, and tolerability of oral treatment with D-mannose versus oral treatment with fosfomycin trometamol in female patients with cystitis. The study duration was eight days (acute phase) with follow-up to day 29.

The study was conducted in accordance with the guidelines of the International Council for Harmonization for Good Clinical Practice and the principles of the Declaration of Helsinki. It was approved by an independent ethics committee, and written informed consent was obtained from all participants. Data were collected in medical practices and clinical trial units in Germany.

### 4.2. Participants and Treatments

Female outpatients aged 18–70 years were eligible if they had clinical signs and symptoms of cystitis with onset not earlier than 72 h prior to enrollment, an ACSS Typical Domain Score of ≥6 at baseline, and leukocyturia at baseline. Key exclusion criteria were episodes of acute cystitis in the four weeks before enrollment or meeting the definition of chronic recurrent cystitis (≥3 episodes in six months), the presence of or predisposition to complicated urinary tract infections, evidence of pyelonephritis, or vulvovaginitis. Details of the inclusion and exclusion criteria, including prior antibiotic use, are provided in the supplement.

Patients were randomized in a 1:1 ratio to receive the investigational treatment of D-mannose or the comparator treatment of fosfomycin. The randomization schedule was generated with SAS 9.4 TS1M7 prior to the start of the investigation by an external service provider. A block randomization scheme with four patients per block (with an allocation ratio of treatment groups 1:1) was applied. A double-dummy technique and identical appearance of all treatment packages (investigational treatment, comparator, placebos) ensured the blinding of the study. Treatment packages were prepared by an external service provider and dispatched to the study sites. Packages were numbered sequentially. Patients, investigators, and on-site study personnel remained blinded throughout the study. Sealed emergency codes made it possible to match randomization numbers and treatment packages only in an emergency.

Patients in the D-mannose group received 2 g of D-mannose for five days (three times daily on days 1–3 and twice daily on days 4–5), plus a single dose of fosfomycin-matching placebo. Patients randomized to the fosfomycin group received a single dose of 3 g fosfomycin trometamol on day 1, plus D-mannose-matching placebo for five days.

### 4.3. Endpoints and Assessments

No primary endpoint was defined for this proof-of-concept study. Efficacy endpoints included change in the ACSS Typical Domain Score up to day 8, the proportion of patients with CC (three definitions) on day 8 and day 29, time to CC within the first eight days, the proportion of patients with UTI recurrence, the proportion of patients requiring additional antibiotic treatment for UTI, and the proportion of patients with a microbiologically negative urine culture on day 8. Safety endpoints included adverse events, as well as investigator and patient global assessments of tolerability on day 8 and day 29. Details of the endpoints are provided in the supplement.

The ACSS Typical Domain Score includes six patient-reported items: urinary frequency, urgency, dysuria, suprapubic pain, incomplete voiding, and visible blood in the urine. Symptom intensity is rated on a four-point Likert scale (0 = none, 1 = mild, 2 = moderate, 3 = severe). A summary Typical Domain Score of ≥6 indicates a clinical diagnosis of cystitis [28,29,31]. The ACSS questionnaire was completed daily in the evening until day 8 and in the follow-up phase if indicated.

### 4.4. Statistical Methods

#### 4.4.1. Sample Size Estimation

Initially, we planned to randomize 436 patients. The sample size was adjusted due to slow recruitment, and a statistical power approach for a proof-of-concept study was incorporated by protocol amendment. The approach assumed that approximately 50 patients per treatment group would provide approximately 70% power to detect one-sided *p*-values ≤ 0.10. Assuming a dropout rate of 15%, 120 patients would be randomized.

#### 4.4.2. Analyses According to Protocol

Efficacy assessments were performed on the PPS, which included all patients in the FAS, i.e., all randomized patients with at least one documented dose of study drug and post-baseline efficacy data who had no relevant protocol deviations and were considered clinically evaluable. The PPS was considered a more conservative analysis population in a non-inferiority setting. To assess the robustness of the efficacy results, sensitivity analysis for clinical cure at day 8 was additionally performed using the FAS. Key efficacy endpoints were analyzed using a non-inferiority testing procedure with a non-inferiority margin of 15% for rate differences at the lower limit of the confidence intervals (95% two-sided Wald confidence intervals). The non-inferiority margin was determined based on the established treatment effect of antibiotic therapy compared with placebo, following the FDA Guidance for Industry on Non-Inferiority Clinical Trials [34]. According to the meta-analysis by Mitrani-Gold et al. [35], the healing rate attributable to antibiotic treatment in previous placebo-controlled UTI trials was estimated, after adjustment for the placebo response, and resulted in an M1 value of 0.32 (>32%). Consistent with FDA recommendations, the largest clinically acceptable difference between the investigational treatment and the active control should preserve at least 50% of the active control treatment effect, resulting in a non-inferiority margin (M2) of ≤15%.

Safety assessments were generally performed on the SEP, which included all randomized patients with at least one documented dose of study drug and a post treatment safety assessment. For tolerability assessments, differences between treatment groups were evaluated using the Wilcoxon rank test adjusted for center and additionally for baseline ACSS severity status, respectively (van Elteren test). A one-sided significance level of 2.5% was used. For numerical data with metric scale, the number of valid observations, arithmetic mean, standard deviation, median, minimum, and maximum values were presented. For categorical data, the absolute numbers (n) and percentages (%) (based on valid cases) were presented.

All per-protocol analyses were performed using SAS version 9.4.

#### 4.4.3. Post Hoc Analysis

Post hoc analysis was performed to quantify and compare the daily changes in cure between treatment groups and to account for the limited sample size as well as possible data bias due to both (the per-protocol replacement of) missing ACSS values and the CC persistence rule defined in the protocol (i.e., once CC was reached on a specific study day, CC was deemed to persist on all following days until recurrence or the last study day). To achieve this adjustment, missing ACSS data were replaced using a multiple imputation procedure. Multiple imputation is a well-known statistical method used to observe and estimate possible effects of the uncomplete data samples based on refined data evaluations (by creating several plausible complete datasets, analyzing each independently, and pooling the results). CC rates (see Figure 2B) were recalculated from the ACSS data included imputed values and the arbitrary persistence rule was not applied in the post hoc analysis.

The model approach recently published by Möllenhoff and Tresch [23] was applied in the post hoc analysis of the time-to-event endpoints. The Möllenhoff model was used to enable the calculation of a rate difference instead of an hazard ratio for the time-to-event data and therefore allowed results applying one common scale (rates). As the other important endpoint (CC rates) is generally calculated on a rate-based approach, the Möllenhoff model permitted comparison of the time pattern including estimation of *p*-values and confidence intervals (even for subsets of days), as well as comparison of treatment effects (e.g., time-to-event and CC), on the same scale, at all time points, and in each model. The results hereof were influenced by the censoring times, which are important in time-to-event analyses. Censoring times are the points when a participant drops out or a study ends (without reaching CC), leaving the true time of CC incompletely known. Therefore, the time-to-event endpoints did not need multiple imputation. The proposed time-to-event models considered two cases (see Figure 2C,D): (a) a nonparametric approach, i.e., no distribution of the data assumed, using the standard Kaplan–Meier estimators (known from survival analysis) and (b) a parametric approach with assumed lognormal distributed data to maximize precision as proposed by the Akaike information criterion (AIC) values.

Additionally, the variances of the parametric model were estimated by bootstrap to enhance the statistical quality of the results. Bootstrap methods allow a more robust inference when theoretical derivations are complex or strongly dependent of model assumptions and cannot be clearly controlled by model assumptions (here, variance estimators and related confidence intervals were used) [23,36]. For both Möllenhoff models, so-called ‘confidence bands’ were constructed for the treatment differences in the time interval considered (days 1 to 8). These bands could be used to test for non-inferiority of D-mannose versus fosfomycin at each time point and for groupings of time points.

The four analyses (see Figure 2) used models of varying levels of complexity to generate results on comparable scales. It was expected that the refined statistical methods of the Möllenhoff models (especially parametric modelling with bootstrap estimates of variance estimators) would increase the precision of the results.

In addition, the ACSS Typical Domain Score after multiple imputations was descriptively investigated for treatment differences. Estimates of UTI recurrence were also investigated for potential bias due to unequal dropout rates and compared by group.

Based on the study data, simulation analyses were performed using both Möllenhoff models to assess whether non-inferiority could have been achieved with higher sample sizes. Post hoc analysis was performed applying the R software (www.r-project.org/, version 3.6.3) and related software packages.

## Figures and Tables

**Table 1:** Descriptive presentation of patient demographic and clinical characteristics at baseline in the safety evaluation population (SEP) by treatment group.

	D-Mannose	Fosfomycin	Statistics
	N = 61	N = 57	*p*-Value
**Age, years**			
Mean (SD)	40.8 (13.9)	41.5 (14.9)	*p* = 0.793 ^b^
Median	40.0	41.0	
Min–Max	21–69	18–70	
**Ethnic origin**			
Caucasian, n (%)	58 (95.1)	57 (100.0)	*p* = 0.245 ^c^
Asian, n (%)	3 (4.9)	0 (0.0)	
**BMI**			
Mean (SD)	24.94 (4.66)	25.56 (5.74)	*p* = 0.523 ^b^
Median	23.46	23.44	
Min–Max	18.3–38.1	17.6–41.6	
**Positive urine culture for bacteria, n (%)**	36 (59.0)	35 (61.4)	*p* = 0.852 ^c^
***E. coli*** **infection, n (%)**	28 (45.9)	29 (50.9)	*p* = 0.713 ^c^
**ACSS Typical Domain Score ^a^**			
Mean (SD)	10.8 (3.0)	10.2 (2.8)	*p* = 0.263 ^b^
Median	11.0	10.0	
Min–Max	6–17	6–16	
**Disease severity by category** **(ACSS Typical Domain Score) ^a^**			
Moderate (6–12), n (%)	41 (67.2)	44 (77.2)	*p* = 0.305 ^c^
Severe (>12), n (%)	20 (32.8)	13 (22.8)	*p* = 0.113 ^d^

^a^ The ACSS Typical Domain Score can range from 0 to 18, ^b^ *t*-test, ^c^ Fisher’s exact test, ^d^ (one-sided) test after arcsin(sqrt(x))-trafo for scores = ‘severe’. *p*-values < 0.05 indicate statistically significant differences (none found).

**Table 2:** Patients with treatment-emergent adverse events during the study in the safety evaluation population (SEP).

	D-Mannose	Fosfomycin	Statistics
	N = 61	N = 57	OR [95% CI] ^a^; *p*-Value
**Overview of patients with treatment-emergent AEs, n (%)**			
Any AEs during the study	20 (32.8)	26 (45.6)	0.58 [0.26, 1.31]; *p* = 0.187
AEs during the acute phase	9 (14.8)	16 (28.1)	0.45 [0.16, 1.20]; *p* = 0.114
SAEs	0 (0.0)	1 (1.8)	n.e.
Severe AEs	0 (0.0)	1 (1.8)	n.e.
AEs leading to treatment discontinuation	2 (3.3)	1 (1.8)	n.e.
AEs leading to study withdrawal	2 (3.3)	1 (1.8)	n.e.
AEs related to study device/drug	6 (9.8)	13 (22.8)	0.37 [0.11, 1.15]; *p* = 0.079
**Patients with AEs by System Organ Class, n (%)**			
Gastrointestinal disorders	6 (9.8)	14 (24.6)	0.34 [0.10, 1.03]; ***p* = 0.048**
General disorders and administration site conditions	2 (3.3)	0 (0.0)	n.e.
Infections and infestations	8 (13.1)	7 (12.3)	1.08 [0.32, 3.77]; *p* = 1.0
Metabolism and nutrition disorders	0 (0.0)	1 (1.8)	n.e.
Musculoskeletal and connective tissue disorders	2 (3.3)	0 (0.0)	n.e.
Nervous system disorders	6 (9.8)	8 (14.0)	0.67 [0.18, 2.38]; *p* = 0.574
Reproductive system and breast disorders	1 (1.6)	1 (1.8)	n.e.
Respiratory, thoracic, and mediastinal disorders	0 (0.0)	2 (3.5)	n.e.

^a^ Exact Fisher-test (OR = odds ratio estimate and 95% CI; p). n.e. (not evaluable) denotes data with totals less than or equal to 3. *p*-values < 0.05 indicate statistical significance (highlighted in bold).

## Data Availability

The data presented in this study are available on request from the corresponding author.

## Supplementary Materials

The following supporting information can be downloaded at https://www.mdpi.com/article/10.3390/antibiotics15090820/s1. In- and exclusion criteria, Endpoints, Clinical Cure (CC) Definition, Definition of recurrence of UTI, Table S1: Descriptive presentation of patient demographic and clinical characteristics, Figure S1: Patient disposition flowchart (all enrolled subjects), Figure S2: Sensitivity analyses of CC at day 8; Figure S3: Treatment differences in CC, Figure S4: Per-protocol analysis of ACSS Typical Domain Score from baseline (V1) to day 8, Figure S5: Global assessment of efficacy on day 8.
