# Supplementary material for: First Randomized Controlled Trial Comparing D-Mannose to Fosfomycin in Acute Uncomplicated Lower Urinary Tract Infections
Source: Antibiotics (Basel). 2026 Aug 24;15(9):820. doi: 10.3390/antibiotics15090820 (PMC13603419; doi:10.3390/antibiotics15090820)
Supplement: Supplementary file 1 [file antibiotics-15-00820-s001.zip › antibiotics-4458218-supplementary.pdf]

Article

First Randomized Controlled Trial Comparing D-Mannose to Fosfomycin in Acute Uncomplicated Lower Urinary Tract Infections

Supplement

1. In- and Exclusion Criteria

1.1. Inclusion Criteria

1. Female outpatients aged 18 to 70 years.
2. Clinical signs and symptoms of an acute uncomplicated cystitis (e.g., dysuria, pollakiuria and/or urgency to urinate) started not earlier than 72 hours prior to study enrolment at visit 1.
3. Signed informed consent and data protection form.
4. ACSS Typical Domain Score was ≥ 6 at visit 1.
5. Urine dipstick test (midstream sample) was positive for leukocytes at visit 1.
6. Valid only for protocol versions 1.0 and 1.1: Urine culture was positive for bacteria defined as ≥ 10^3 colony-forming units per mL urine of single or mixed culture of uropathogens (mixed = maximum of two species). Criterion 6 was deleted from protocol version 1.2 as the ACSS questionnaire is a fully validated instrument to confirm the diagnosis of cystitis.

1.2. Exclusion Criteria

1. Males.
2. Pregnant or lactating females or females of childbearing potential who were not using an effective method of contraception.
3. Females with episodes of acute cystitis within four weeks before enrolment (visit 1) or females with ≥ 3 episodes of acute cystitis within six months before enrolment.
4. Anamnestic proof of presence of any predisposing factors or any conditions that could promote or lead to (complicated) urinary tract infections (i.e., anamnestic proof of renal diseases, renal calculi, polycystic kidneys, urinary tract abnormalities or past urinary surgery, urine catheterization, spinal cord injury, etc.).
5. Anamnestic proof of renal impairment (creatinine clearance < 30 mL/min).
6. Anamnestic proof of diabetes mellitus (Type I and II).
7. Any signs of pyelonephritis (i.e., fever ≥ 38°C, tympanic, flank and/or back pain, chills, and shivers).
8. Vulvovaginitis with abnormal vaginal and/or urethral discharge (without urination) at visit 1.
9. Known or suspected hypersensitivity to one or more of the ingredients of the test product (Femannose® N) and/or comparator (Fosfomycin HEXAL®) and/or any placebo used.
10. Systemic or local (i.e., vaginal) treatment with antibiotics within seven days prior to visit 1.
11. Treatment with analgesics (e.g., nonsteroidal anti-inflammatory drugs, etc.) within six hours prior to visit 1.
12. Parallel participation in another clinical study or study, participation in a clinical study or study within the previous four weeks prior to visit 1, or previous participation in this clinical study.
13. Known to be or suspected of being unable to comply with the study protocol (e.g., no permanent address, prior or current drug abuse, known to be non-compliant or presenting an unstable psychiatric history).
14. Legal incapacity and/or other circumstances which made the subject unable to understand the nature, scope, and possible impact of the investigation.
15. Custody by juridical or official order.
16. Evidence of an uncooperative attitude (non-compliance).
17. Difficulties in understanding the language in which the informed consent and data protection declaration was given.

18. Investigational site staff, staff employed by the sponsor or involved clinical research organization (CRO), the investigator him-/herself, or close relatives.

2. Endpoints

2.1 Efficacy Endpoints

1. Percentage of subjects with clinical cure (CC) on either day 8 (visit 2) or day 29 (visit 3) and on both days.
2. Time to first day of CC within the first eight days of the active phase of the study.
3. Percentage of subjects with a negative urine culture at visit 2.
4. Percentage of subjects with CC and a negative urine culture at visit 2.
5. Percentage of subjects with CC and ACSS QoL Domain Score ≤ 3 but no QoL domain item > 1 on day 8 (at visit 2).
6. Percentage of subjects with recurrence of UTI established by ACSS Typical Domain Score ≥ 6 during the follow-up phase.
7. Percentage of subjects undergoing antibiotic therapy for acute UTI in the acute phase (visit 1 to visit 2, in addition to the study treatment), in the follow-up phase (visit 2 to visit 3: recurrence by antibiotic therapy), and in both study phases (visit 1 to visit 3).
8. Time to recurrence of UTI as determined by the first date the ACSS Typical Domain Score is ≥ 6 during the follow-up phase.
9. Time to recurrence of UTI as determined by the first date of antibiotic use for acute UTI during the follow-up phase.
10. Change in ACSS Typical Domain Score on day 8 compared to visit 1.
11. Change in ACSS QoL Domain Score on day 4 and day 8 each compared to visit 1.
12. Change in the total score calculated as sum of the two ACSS Typical and ACSS QoL Domain Scores on day 8 compared to visit 1.
13. Change in time course of the ACSS Dynamic Domain Score from day 1 to day 8.
14. Investigators' and subjects' global judgement on efficacy at visit 2 and visit 3.

2.2. Safety Endpoints

1. Adverse events.
2. Change in vital signs at visit 2 and visit 3, both compared to visit 1.
3. Investigators' and subjects' global judgement on tolerability at visit 2 and visit 3.
4. Percentage of subjects with co-medication at visit 2 and visit 3, compared to visit 1.

3. Clinical Cure (CC) Definition

- Main definition: ACSS Typical Domain Score ≤ 4 (sum of the six scores within the domain was ≤ 4, whereby items one to five had to be scored '1' (mild) or '0' (not present), and item six had to be scored '0' (absence of hematuria, i.e., no visible blood in the urine).
- Variation 1: Summary score within ACSS Typical Domain for the items 'frequency', 'urgency', 'dysuria', and 'suprapubic pain' ≤ 4 with each item scored ≤ 1, and absence of hematuria (sum of four scores within the domain; used only for Endpoint 1 analysis).
- Variation 2: Summary score within ACSS Typical Domain for the items 'frequency', 'urgency', and 'dysuria' ≤ 3 with each item scored ≤ 1, and absence of hematuria (sum of three scores within the domain; used only for Endpoint 1 analysis).

Once CC was reached on a specific study day, CC was deemed to persist on all following days until recurrence of UTI was observed or until the last day of the study. This persistence rule was not applied for post hoc analysis.

For combined endpoints, the combination of an indeterminate outcome and a response outcome resulted in an indeterminate outcome of the respective combined endpoint. The combination of a non-response outcome and an indeterminate outcome resulted in a non-response outcome.

4. Definition of Recurrence of UTI

Recurrence of UTI presented in the manuscript was defined as follows:

- Established by ACSS Typical Domain Score ≥ 6 during the follow-up phase (starting the day after visit 2) and patient had previously been in CC.
- Antibiotics required for acute UTI treatment during acute and follow-up phase, i.e., between visit 1 (day 1 onsite) and visit 3.

5. Patient Disposition

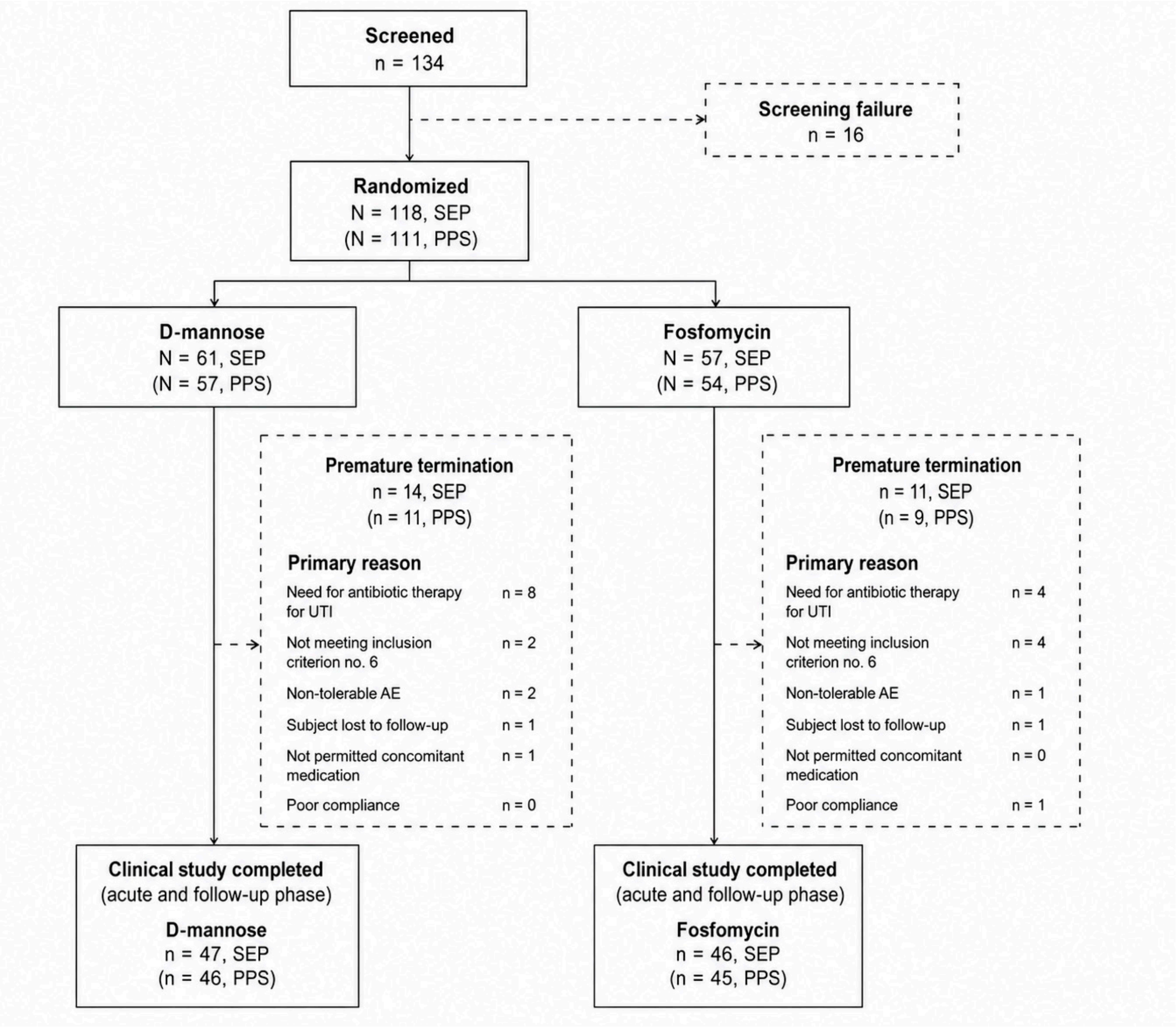

Figure S1. Patient disposition flowchart (all enrolled subjects).

6. Supplementary Tables

Table S1. Descriptive presentation of patient demographic and clinical characteristics at baseline in the per-protocol set (PPS) by treatment group.

	**D-mannose**	**Fosfomycin**
	**N = 57**	**N = 54**
**Age, years**		
Mean (SD)	41.0 (13.9)	40.9 (15.1)
Median	40.0	40.5
Min–Max	21–69	18–70
**Ethnic origin**		
Caucasian, n (%)	54 (94.7)	54 (100.0)
Asian, n (%)	3 (5.3)	0 (0.0)
**BMI**		
Mean (SD)	25.25 (4.67)	25.47 (5.88)
Median	23.80	23.38
Min–Max	18.3–38.1	17.6–41.6
**Positive urine culture for bacteria, n (%)**	34 (59.6)	33 (61.1)
***E. coli* infection, n (%)**	26 (45.6)	27 (50.0)
**ACSS Typical Domain Score** [a]		
Mean (SD)	10.8 (3.0)	10.2 (2.9)
Median	11.0	10.0
Min–Max	6–17	6–16
**Disease severity by category (ACSS Typical Domain Score)** [a]		
Moderate (6–12), n (%)	39 (68.4)	41 (75.9)
Severe (> 12), n (%)	18 (31.6)	13 (24.1)

7. Supplementary Figures

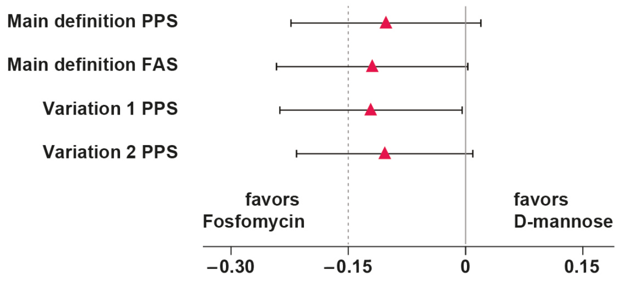

Figure S2. Main and sensitivity analyses of treatment differences in clinical cure (CC) at day 8. Red markers indicate point estimates of treatment differences, bars indicate 95% CI. The dashed line indicates the non-inferiority margin (for details on main definition and variations see section 3).

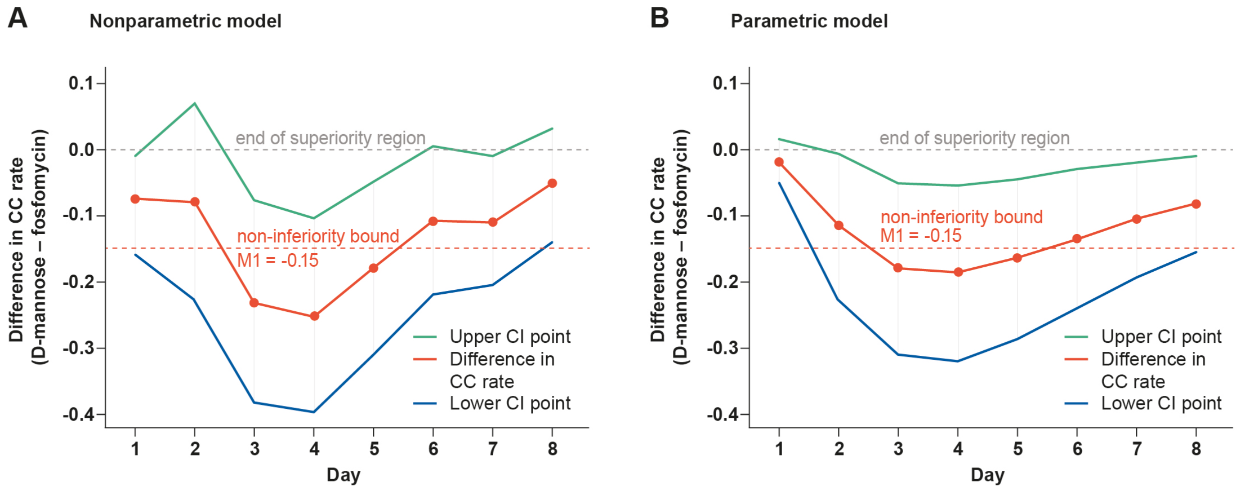

Figure S3. Treatment differences in clinical cure (CC) with 95% confidence bands from day 1 to day 8 in the per-protocol set (PPS) based on the post hoc analysis of CC data according to the nonparametric (A) and parametric (B) Möllenhoff model. Red lines with red dots indicate point estimates of treatment differences, green lines indicate the upper 95% CI boundary, blue lines indicate the lower 95% CI boundary. The red dotted lines indicate the non-inferiority margin M1 of -0.15.

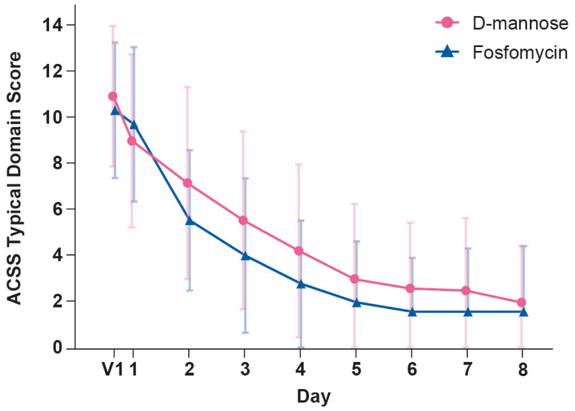

Figure S4. Per-protocol analysis of ACSS Typical Domain Score from baseline (V1) to day 8 in the PPS. Markers indicate the means. Error bars indicate the standard deviations.

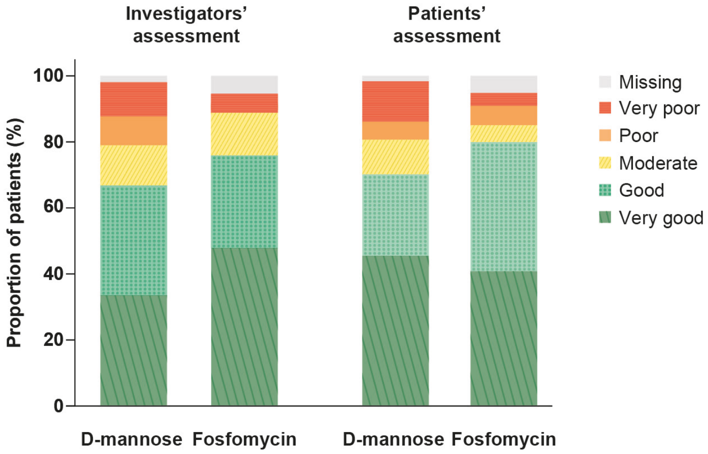

Figure S5. Global assessment of efficacy on day 8 in the PPS. Missing data at visit 2 was imputed with the worst category ('very poor') for withdrawals due to the need for antibiotic therapy for urinary tract infection, which is associated with a lack of efficacy. All other missing data not associated with a lack of efficacy are summarized under the category 'missing/dropout'.
